# Efficacy and safety of *Onkyeong-tang* in treating cold hypersensitivity in the feet of Korean women: protocol for a double-blind, randomized, placebo-controlled, parallel-group, multicenter clinical study

**DOI:** 10.1186/s13063-020-04265-7

**Published:** 2020-05-18

**Authors:** Kyou-Young Lee, In-Sik Han, Ho-Yeon Go, Dong-Nyung Lee, Jun-Sang Yu, Seung-Ho Sun

**Affiliations:** 1 Department of Ophthalmology, Otolaryngology, Dermatology, College of Korean Medicine, Sangji University, 80 Sangjidae-gil, Wonju-si, Gangwon-do Republic of Korea; 2 Department of Korean Internal Medicine, College of Korean Medicine, Sangji University, 80 Sangjidae-gil, Wonju-si, Gangwon-do Republic of Korea; 3 Department of Korean Internal Medicine, College of Korean Medicine, Semyung University, 65 Semyeong-ro, Jecheon-si, Chungcheongbuk-do Republic of Korea; 4 Department of Gynecology Medicine, College of Korean Medicine, Semyung University, 63 Sangbang 4-gil, Chungju-si, Chungcheongbuk-do Republic of Korea; 5 Department of Sasang Constitutional Medicine, College of Korean Medicine, Sangji University, 80 Sangjidae-gil, Wonju-si, Gangwon-do Republic of Korea

**Keywords:** Herbal medicine, Cold stress test, Cold hypersensitivity, *Onkyeong-tang*, Randomized clinical trial

## Abstract

**Background:**

Cold hypersensitivity in the hands and feet (CHHF) commonly affects Asian women, especially Korean women, and it negatively impacts the quality of life of the affected individuals. One commonly used herbal prescription for treating CHHF is *Onkyeong-tang* (OKT). Although OKT is widely used clinically in treating CHHF, no randomized clinical trial has been performed to evaluate the efficacy and safety of OKT in the treatment of cold hypersensitivity in the feet (CHF). This clinical trial aims to provide objective evidence for the basis of using OKT in the treatment of CHF in Korean women.

**Methods:**

This trial will be a double-blind, randomized, placebo-controlled, parallel-group, multicenter pilot study. A total of 112 participants will be randomly divided into an OKT treatment group or a placebo group with a 1:1 ratio via a web-based randomization system. The OKT and placebo groups will receive prescribed medications orally three times per day (3 g each time) before or between meals for 8 weeks. The primary outcome studied will be the changes in Visual Analog Scale (VAS) scores of CHF from baseline. Secondary outcomes studied will be the VAS score changes of cold hypersensitivity in the hands, changes in the skin temperature of the hands and feet, total scores of the Korean version of the World Health Organization Quality of Life Scale-abbreviated version, and the results of the cold stress test.

**Discussion:**

This trial will be the first clinical trial to assess the efficacy and safety of OKT in the treatment of CHF. We anticipate that the findings of the study will provide evidence for the basis of using OKT in treating CHF symptoms and generate basic data for designing a further large-scale randomized clinical trial.

**Trial registration:**

Clinical Research Information Service (CRIS): KCT0003723. Retrospectively registered on 8 April 2019.

## Background

Cold hypersensitivity in the hands and feet (CHHF) is defined as “a sensation of coldness in the hands and feet in an environment not considered cold by unaffected people or having a heightened cold sensation in a relatively low temperature area” or “answering ‘cold’ to both are your hands cold or warm? and are your feet cold or warm?” in a previous study [1]. CHHF commonly affects Asian women, especially Koreans, and it negatively impacts quality of life. The prevalence of CHHF has not been investigated in detail, possibly due to the ambiguous diagnostic criteria of the disease. A questionnaire-based analysis revealed that about 38.7% of 318 women in a study experienced cold hypersensitivity [2].

CHHF is typically defined as a subjective symptom, and most patients with CHHF do not present any obvious underlying physiological causes. To treat CHHF, conventional medicines focus on lifestyle management; however, Korean medicine (KM) considers CHHF a key factor for cold pattern identification and offers treatments such as moxibustion and acupuncture, along with various Korean herbal medicines [3–6]. *Onkyeong-tang* (OKT) is one of the commonly used herbal prescriptions for treating CHHF.

The use of OKT dates back to the beginning of the third century, as described in the *Synopsis of Prescriptions of the Golden Chamber*, a classic Chinese medical book written by Zhang Zhongjing. OKT was also introduced with the name *Chokyungsan* in the classic Korean medical book titled *Treasured Mirror of Eastern Medicine* and written by Heo Jun [7]. This herbal formula is used to treat gynecological diseases such as menstrual irregularities, vaginal discharge, uterine bleeding, menopausal disorder, hypoplasia of the uterus, sterility, as well as other conditions such as frostbite and diarrhea [8]. OKT is also used for treating symptoms which result from the deficiency cold of the thoroughfare and conception vessels by warming the meridian to dissipate cold, activate the blood, and also resolve stasis, nourish the blood, and regulate menstruation [9].

Although OKT is widely used in the treatment of CHHF, no randomized clinical trial (RCT) has yet been conducted to evaluate the efficacy and safety of OKT in the treatment of cold hypersensitivity in the feet (CHF). This double-blind, randomized, parallel-group, placebo-controlled, multicenter clinical trial will aim to determine the effects of OKT in patients with CHF. The findings of this study should provide objective evidence from which to evaluate the basis of the clinical use of OKT in the treatment of CHF and provide valuable information to design a large-scale RCT in the future.

## Methods

### Objectives

This study aims to determine the efficacy and safety of OKT in Korean female patients with CHF compared with a placebo group following 8 weeks of administration.

### Study design and setting

This randomized, double-blind, multicenter, parallel-group, placebo-controlled pilot study will be conducted at the Korean Medical Hospital of Sangji University and Semyung University Korean Medical Hospital at Chungju and Jecheon.

A total of 112 participants will be enrolled in this study. The study and control groups will have 56 participants each, and the participants will be registered competitively in the three centers. An adequate explanation and time will be given to all participants so that they can make a thoughtful decision before the principal investigator (PI) or researchers obtain written informed consent. Each participant will undergo a screening period 2 to 7 days prior to randomization. During the screening period, the following will be performed: measurement of vital signs, body index (height, weight, and body mass index [BMI]), body temperature, and CHHF Visual Analog Scale (VAS) score; laboratory tests, chest X-ray, and electrocardiogram (ECG); identifying the reason and method of study participation and collecting demographic, sociological, and gynecological information; evaluation of general physical state; Questionnaire of Pattern Identification; and determining if the potential participant meets the inclusion criteria. Those who meet the inclusion criteria will be invited to take part in the study. Subsequently, participants will be randomly assigned to the OKT or placebo group.

**Fig. 1:**
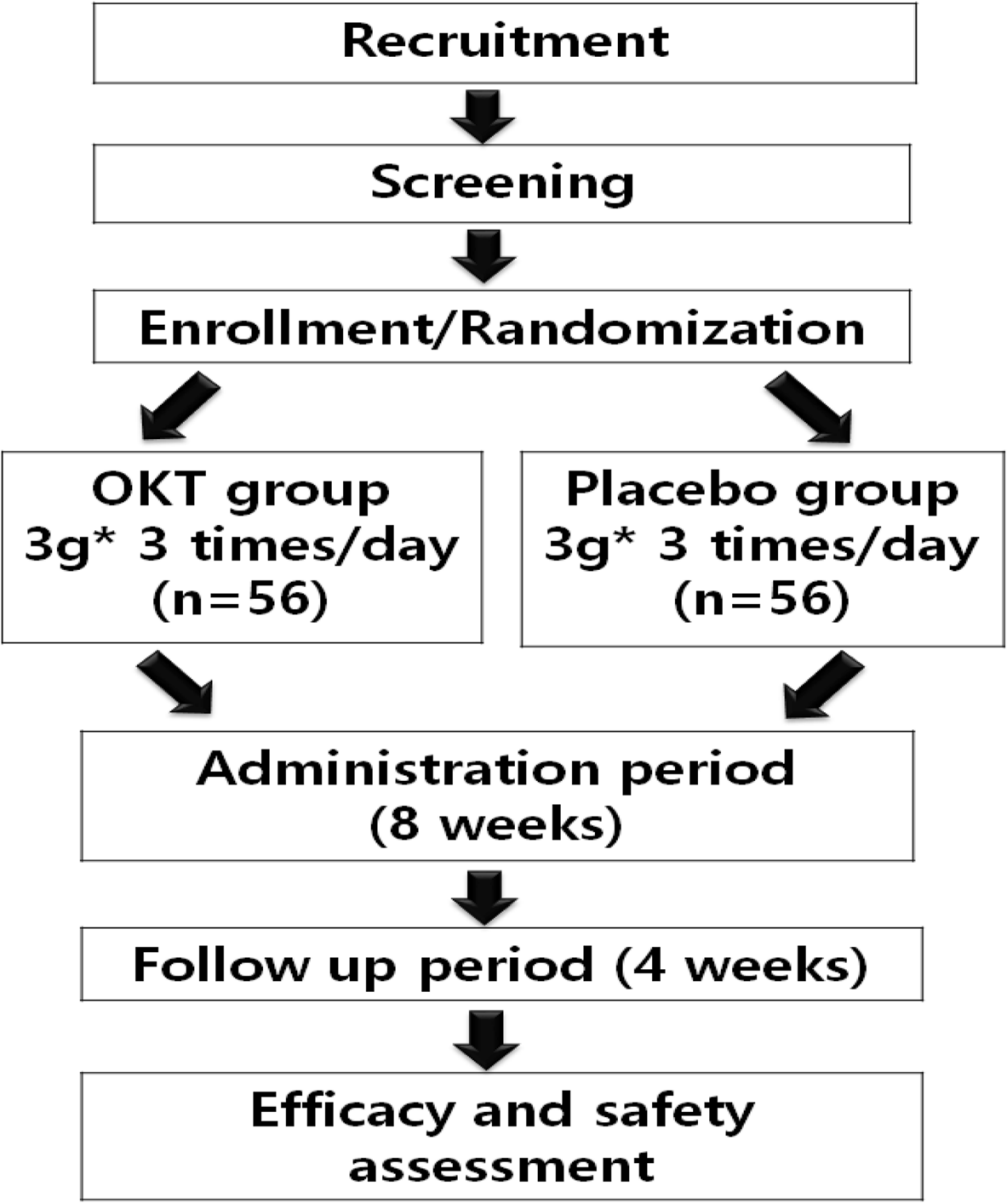
Flow chart of the *Onkyeong-tang* (OKT) study

After randomization, all participants will be asked to visit the center every 4 weeks for 12 weeks, and the investigational product (IP) will be administered for 8 weeks. One follow-up visit will be conducted 4 weeks after the completion of medication. A total of five visits will be scheduled over the span of 13 weeks. At visits 3 and 4, the participants will be asked to return any IPs not taken for the purpose of calculating drug compliance. Participants will be asked not to take other medications which could affect the CHF symptoms during the trial (e.g., antithrombotic drugs, psychotropic drugs, antidepressant drugs, drugs for hyperthyroidism, functional health foods such as red ginseng, and blood flow-enhancing over-the-counter drugs). The protocol design is based on the Standard Protocol Items: Recommendations for Interventional Trials (SPIRIT) Checklist (see Additional file 1). Figure 1 shows a schematic flow diagram of the study.

### Randomization

Eligible participants who met the inclusion criteria will be assigned to a treatment group (OKT) or a placebo group with a 1:1 ratio. An independent statistician from the contract research organization (CRO), the Korean Medicine Clinical Trial Center (K-CTC), will perform the randomization. Random assignment numbers will be generated using a web-based randomization system (blockrand package, version 1.3; Intermountain Healthcare, Greg Snow, USA) and will be stratified by hospital. The randomization table of the participants will be retained by the CRO in an opaque sealed envelope throughout the clinical trial period.

### Blinding

The test medication (OKT) and placebo will be made to appear identical and labeled with the randomization code number. Independent pharmacists or research assistants will administer the trial medications by matching the randomization code numbers to the patients. Clinical trial staff (investigator, research assistants, pharmacist, clinical research coordinator [CRC], clinical research associate [CRA]) and the participants will be blinded to the treatment allocation during the trial. After the end of the trial, the blind will be released for analysis of the results. However, in severe medical emergencies such as serious adverse events (SAEs) during the clinical trial, unblinding may be considered according to the standard operating procedures (SOPs).

### Participants

#### Inclusion criteria

The inclusion criteria are described in Table 1.

**Table 1:** Inclusion criteria for the *Onkyeong-tang* (OKT) clinical trial

Inclusion criteria	
1. Female patients between the ages of 19 and 59 years with a complaint of cold hypersensitivity in the feet (CHF)	
2. Patients must meet at least one or more of the following conditions:	
2.1. Symptoms of CHF at a temperature at which most individuals do not feel cold 2.2. Symptoms of severe CHF during exposure to colder than normal temperature 2.3. Persistent CHF symptoms even after returning to a warmer environment	
3. The patient’s Visual Analog Scale score for CHF should be 4 or more	
4. Thermal deviation between the foot (acupuncture point, LR3) and thigh (acupuncture point, ST32) should be higher than 2 °C when both the lower legs are exposed to room temperature (24 °C ± 2)	
5. Patients should be able to give informed consent	

#### Exclusion criteria

The exclusion criteria are listed in Table 2.

**Table 2:** Exclusion criteria for the *Onkyeong-tang* (OKT) clinical trial

Exclusion criteria	
1. Those who are taking calcium antagonists or beta-blockers as a treatment for CHHF	
2. Those who are experiencing gangrene or ulceration in one or more fingers	
3. Those who are diagnosed with hyperthyroidism or are currently medicated with thyroid drugs	
4. Those who are diagnosed with autoimmune disease(s)	
5. Those who are diagnosed as having tarsal tunnel syndrome or a positive Tinel’s sign and Phalen’s test	
6. Those who are diagnosed with cervical disc herniation	
7. Those who are diagnosed with diabetes	
8. Those who are taking a drug that may influence CHF (e.g., anticoagulants)	
9. Those with a moderate level of liver dysfunction (aspartate aminotransferase [AST] and alanine aminotransferase [ALT] levels both greater than 100 IU/L) or kidney dysfunction (creatinine [Cr] level greater than 2 mg/dL)	
10. Those unable to give informed consent	
11. Non-pregnant adult women with hemoglobin (Hb) level less than 7 g/dL, hematocrit level less than 26%, and white blood cell (WBC) count greater than 11,000/mm^3^	
12. Those whose average systolic blood pressure (SBP) is 180 mmHg or more or whose diastolic blood pressure (DBP) is 100 mmHg or more when measured twice	
13. Those with suspected arrhythmia, as shown with an electrocardiogram (ECG), or those diagnosed with heart disease, such as ischemic heart disease	
14. Those who are addicted to alcohol or drugs	
15. Those who are pregnant (positive urine human chorionic gonadotropin [hCG]), lactating, or planning for pregnancy	
16. Those diagnosed with malignant tumors	
17. Those taking part in other clinical trials	
18. Those who are unable to understand or speak Korean	
19. Those who are judged to be unfit for the clinical trial by the researchers	

#### Withdrawals

Subjects who meet any of the criteria shown in Table 3 will be withdrawn from the study. Reasons for withdrawal will be recorded in a case report form (CRF), and the patient(s)’ data will be analyzed using the intention-to-treat (ITT) method.

**Table 3:** Withdrawal criteria for the *Onkyeong-tang* (OKT) clinical trial

Withdrawal criteria	
1. Those whose compliance is less than 70%	
2. Those who become pregnant during the trial period	
3. Those who need surgery or hospitalization due to accidents or other diseases	
4. Patient withdrawal of consent	
5. Those who have used forbidden medicines or treatments such as anticoagulants, psychotropic drugs, or other medications or treatments that may have effects on the symptoms of CHF	
6. Those who need standard treatment because of the aggravation of CHF symptoms	
7. Occurrence of SAEs	
8. Occurrence of other inevitable situations that may make it difficult to continue the trial process 9. Principal investigator’s (PI’s) judgement to discontinue the trial due to some factors affecting the study results	

### Procedure

#### Recruitment

A total of 112 subjects who meet the inclusion criteria will be competitively recruited through three Korean medicine university hospitals located in three cities in Korea. The Korean Medical Hospital of Sangji University will recruit 32 subjects, Semyung University Korean Medical Hospital at Chungju will recruit 40 subjects, and Semyung University Korean Medical Hospital at Jecheon will recruit 40 subjects. Each institute will recruit subjects through public outdoor advertisements using posters and other means. Those who are interested in participating in the clinical trial will be invited to contact the CRC or PI to obtain detailed information about the clinical trial and to schedule a screening visit.

#### Study schedule

The items to be checked at each patient visit are shown in Table 4.

**Table 4:** Study schedule for the *Onkyeong-tang* (OKT) clinical trial

	Screening	Treatment period			Follow-up
	Visit 1 (day − 7 through − 2)	Visit 2 (day 0)	Visit 3 (day 28 ± 3)	Visit 4 (day 56 ± 3)	Visit 5 (day 84 ± 3)
Informed consent	●				
Eligibility criteria	●				
Randomization		●			
Medication compliance			●	●	
Vital signs	●	●	●	●	●
Body measurements^a^	●	●	●	●	●
Collection of demographic, sociological,^b^ and gynecological information	●				
Medical history	●	●	●	●	●
General physical examination	●	●	●	●	●
Thermometer measurement^c^	●	●	●	●	●
VAS	●	●	●	●	●
Monitoring of AE			●	●	●
Questionnaire of Pattern Identification	●				
Questionnaire of Cold Hypersensitivity		●			
Cold stress test		●		●	
WHOQOL-BREF		●		●	
Laboratory tests^d^	●			●	
Chest X-ray & ECG	●				
Medication		●	●		
Test of blindness				●	

^a^ Height, weight, and BMI, but only weight for visits 2, 3, 4, and 5

^b^ Age, occupation, digestion, exercise, smoking, drinking, sleep, etc.

^c^ Thermometer measurement of ST32, LR3, PC8, and LU4 at every visit

^d^ Screening: Hematological examination (WBCs, RBCs, Hb, platelets), blood chemistry test (BUN, Cr, AST, ALT, r-GTP, glucose), thyroid function test (free T4, TSH), urine test, pregnancy test (urine hCG)

Visit 4: Hematological examination (WBCs, RBCs, Hb, platelets), blood chemistry test (BUN, Cr, AST, ALT, r-GTP)

Abbreviations: *AE* adverse event, *ALT* alanine aminotransferase, *AST* aspartate aminotransferase, *BMI* body mass index, *BUN* blood urea nitrogen, *Cr* creatinine, *ECG* electrocardiogram, *r-GTP* gamma-glutamyl transpeptidase, *Hb* hemoglobin, *hCG* human chorionic gonadotropin, *RBC* red blood cell, *TSH* thyroid-stimulating hormone, *VAS* Visual Analog scale, *WBC* white blood cell, *WHOQOL-BREF* World Health Organization Quality of Life Scale-abbreviated version

### Interventions

After enrollment, the participants will receive 3 g of OKT or placebo three times a day for 8 weeks. Each group will take the designated medicine with warm water before or between meals.

The OKT and placebo will be supplied by Hanpoong Pharm & Foods Co. Ltd, Jeonju, Republic of Korea. We will be using OKT granulated extract which is composed of 1 g of *Evodiae Fructus*, 0.67 g of *Ginseng Radix Alba*, 0.67 g of *Paeoniae Radix*, 0.67 g of *Asini Corii Colla*, 0.67 g of *Cnidii Rhizoma*, 1.67 g of *Pinelliae Tuber*, 0.67 g of *Moutan Cortex Radicis*, 3.33 g of *Liriopis Tuber*, 1 g of *Angellicae Gigantis Radix*, 0.33 g of *Zingiberis Rhizoma*, and 0.67 g of *Glyzzirizae Radix*. These herbal medicines will be extracted and concentrated to 3 g per dose. The placebo granulated extract is composed of 1.7 g of lactose, 1 g of corn starch, 0.1 g of citric acid, 0.1 g of caramel coloring, and 0.1 g of *Ssanghwa* herbal flavor per dose. The placebo will appear identical to OKT in terms of color, shape, weight, flavor, and taste.

### Outcomes

The primary outcome will be changes in the CHF VAS score from baseline. Secondary outcomes will be the changes in VAS scores for cold hypersensitivity in the hands (CHH), changes in body skin temperature (BT) of the hands and feet, total score of the Korean version of the World Health Organization Quality of Life Scale-abbreviated version (WHOQOL-BREF), and the results of the cold stress test (CST).

#### Primary outcome variable

The primary outcome variable is VAS measurement of CHF symptoms. The VAS score is a 10-point scale representing no coldness as 0 and most severe coldness as 10. It will be used to evaluate the severity of CHF symptoms. The VAS will be measured at every visit, and the changes in score from baseline will be calculated.

#### Secondary outcome variables

##### VAS: measurement of CHH symptoms

The CHH VAS score will be used to assess the severity of symptoms. It will be measured at every visit, and the changes in score from baseline will be calculated.

##### BT: thermometer measurement

Participants will be asked to refrain from consuming caffeine, alcohol, and cigarettes; performing strenuous exercise; and taking hot showers at least 2 h prior to measurement. After a relaxation period of 20 min at 24 ± 1 °C in the examination room, the BT of both palms (PC8), anterior upper arms (LU4), center of the front thighs (ST32), and junctions of the first and second metatarsal bones (LR3) will be measured using a thermometer (Testo 835-T1; Testo, Lenzkirch, Germany) at every visit.

##### WHOQOL-BREF

The WHOQOL-BREF is a questionnaire used to assess quality of life based on 26 items. It consists of five domains: general quality of life, physical health, psychological health, social relationships, and environmental health. The Korean version of the WHOQOL-BREF developed by Min et al. [10] will be used to assess the quality of life of the patients with CHF symptoms at visits 2 and 4.

##### CST

The purpose of the CST is to measure the temperature recovery ability of both feet after cold stimulation. In this procedure, first the participants will rest for 20 min in a room set at 24 ± 1 °C to allow for stabilization to occur, and then the temperature of both feet will be measured. Next, participants will immerse both feet into a container filled with water at 20 °C for 30 s. Subsequently, their feet will be carefully dried with a towel, and the temperature of both feet will be measured immediately and then 6 min later. The temperature measurement point will remain the same for each foot. BT will be measured using an infrared thermometer (Testo 835-T1). The CST will be conducted at visits 2 and 4. The following is the recovery rate (RR) equation for the CST. The change in RR from baseline will be measured.

$$ \mathrm{RR}=\left({T}_6-{T}_0\right)/\left({T}_{base}-{T}_0\right)\times 100\%, $$

where:

*T*_6_ is the skin temperature 6 min after the CST

*T*_0_ is the skin temperature immediately after the CST

*T*_base_ is the skin temperature at baseline

### Questionnaire of Pattern Identification, Questionnaire of Cold Hypersensitivity

The Questionnaire of Pattern Identification will show the distribution of the pattern identification index in patients with CHF and will be performed at visit 1. The Questionnaire of Cold Hypersensitivity will be performed at visit 2.

### Safety assessment

To assess safety, vital sign measurement and a general physical examination will be performed at each visit. Renal and liver function tests (blood urea nitrogen [BUN], creatinine [Cr], aspartate aminotransferase [AST], alanine aminotransferase [ALT], and gamma-glutamyl transpeptidase [r-GTP]) and hematological examinations (white blood cells [WBCs], red blood cells [RBCs], hemoglobin [Hb], and platelets) will be conducted at visits 1 and 4. Confirmation of adverse events [AEs] will be performed at visits 3, 4, and 5.

### Compliance calculation

The participants will be provided with a total of 93 investigational medications at visits 2 and 3. Compliance calculation will be conducted at visits 3 and 4. The formula for calculating medication compliance is as follows:

Compliance (%) = (93 – remaining drugs/expected intake) × 100.

### Adverse event reporting

The PI will educate the co-investigator and the participants about any AEs that may occur after administration of the study drug, and also instruct them to report all symptoms that may occur after administration. The type, occurrence time, extent, care, treatment medicine, progress of symptoms after using the research medicine, and causal relationship with the study drug should be noted in the CRF. If SAEs occur, the site investigator should notify the Institutional Review Board (IRB) and PI. After receiving the SAE report, the site PI will decide whether the participant should be withdrawn or continue to participate in the clinical trial.

### Sample size calculation

This study was designed based on the assumption that when participants with CHF take OKT, their symptoms would be improved compared to the symptoms of participants who take the placebo. The hypothesis based on this assumption is as follows:

$$ \mathrm{H}0:\updelta =\Delta 1-\Delta 2=0 $$

$$ \mathrm{H}1:\updelta =\Delta 1-\Delta 2\ne 0, $$

where:

Δ1 is the average change in VAS score 8 weeks after randomization (within ±3 days) in the OKT group

Δ2 is the average change in VAS score 8 weeks after randomization (within ±3 days) in the placebo group

The sample size calculation was based on the VAS score information of a previously reported clinical trial involving red ginseng [11]. Considering a dropout rate of 20%, we will need to recruit 56 participants in each group to measure the therapeutic effects and to inform the power calculation for a large-scale RCT of OKT in the future.

### Statistical analysis

The basic statistical analysis method will be the degree of change in the efficacy assessment index between the results of the baseline tests performed before the administration of the test drug and the results of the tests at the end of treatment. The statistical significance level will be set at *P* < 0.05. SPSS for windows version 25.0 (IBM Inc., Chicago, IL, USA) will be used for performing the statistical analysis.

#### General characteristics

To test whether the distributions of variables in the test and control groups are homogeneous, an analysis of variance (ANOVA) or non-parametric methods will be used for continuous variables, and chi-squared tests will be applied for categorical variables.

#### Efficacy

For the efficacy assessment, ITT analysis, a method for including all randomly assigned patients in the analysis regardless of violation or dropout, will mainly be used. Per-protocol (PP) analysis, a method which excludes subjects who do not comply with the study protocol or who have missing data, will also be implemented. The missing values of the efficacy assessment variables will be analyzed using the last-observation-carried-forward (LOCF) method.

A paired *t* test will be used for within-group analysis of the primary and secondary outcome variables. Between-group analysis will be performed using repeated measures ANOVA and the paired *t* test for the primary outcome variable. To measure secondary outcome variables, mean changes in the CHH VAS score, the BT, and the results of the WHOQOL-BREF and CST will be assessed. Repeated measures ANOVA and paired *t* tests will be carried out for the CHH VAS score. A repeated measures ANOVA will also be performed for BT. ANOVA and post hoc analyses will be performed for the WHOQOL-BREF results, and a paired *t* test will be used for the CST results. For the analysis of the Questionnaire of Pattern Identification, ANOVA and post hoc tests will be performed, and for the Questionnaire of Cold Hypersensitivity, Cronbach’s alpha and a correlation analysis will be performed.

### Safety

For the clinical laboratory test data, depending on the nature of the variables, ANOVA, *t* test, chi-squared, or Fisher’s exact test will be conducted. The 95% confidence interval for the number of AEs and the percentage of patients who may experience one or more AEs will be calculated within groups and compared between groups. In the case of safety assessment variables, the missing values will be analyzed as missing.

### Data management and monitoring

Paper CRF files will contain all records. The CRF files will be stored in a securely locked location. Participant identification and privacy information will be deleted from all study documents to protect confidentiality. Once the trial is completed, a double independent data entry will be conducted to promote data quality. After the data entry is finished, the database will be locked and analyzed by an independent statistician under the supervision of the PI. Site investigators will have the right to directly access the final datasets from their own sites.

According to the guidelines for establishing and operating an Independent Data Monitoring Committee (IDMC), written by the Korean Ministry of Food and Drug Safety (MFDS) [12], based on guidelines by the US Food and Drug Administration (FDA), World Health Organization (WHO), and Europe, the Middle East, and Africa (EMEA) in Europe, not all clinical trials require an IDMC. The effectiveness of an IDMC is very low in clinical trials in non-fatal indications, where patients are treated for relatively short periods of time and the characteristics of the drug being tested are well defined and known not to cause harm to the patient. In this clinical trial, OKT will be administered orally for a short period of 8 weeks, and the OKT product was already approved for use for coldness of the lower limbs. Thus, it was determined that an IDMC is not needed. Nevertheless, instead of an IDMC, the K-CTC will monitor the institutions conducting the clinical trial based on the SOPs while the trial is in progress to control data quality. When the first patient finishes the necessary visits, the monitoring will begin. Double data entry and range checks for data values will be conducted to improve the quality of data. No auditing is scheduled for this study.

## Discussion

From the perspective of conventional medicine, CHHF presents clinical symptoms of Raynaud’s phenomenon (RP), which is categorized as a peripheral vascular disease. Therefore, patients with CHHF are mostly prescribed vasodilators or antihypertensive drugs. In contrast, in Eastern medicine, coldness is considered as one of the major pathogenic factors. Therefore, the primary treatment principle of CHHF in Eastern medicine is to balance the Yin and Yang energy of the body with personalized and holistic approaches [13].

OKT was named for its warming, tonifying, enriching, and nourishing effects [9]. It consists of 11 components, including *Evodiae Fructus*, *Ginseng Radix Alba*, *Paeoniae Radix*, *Asini Corii Colla*, *Cnidii Rhizoma*, *Pinelliae Tuber*, *Moutan Cortex Radicis*, *Liriopis Tuber*, *Angellicae Gigantis Radix*, *Zingiberis Rhizoma*, and *Glyzzirizae Radix*. Among these, *Ginseng Radix Alba*, *Asini Corii Colla*, and *Liriopis Tuber* are used to tonify Qi, engender fluids, and tonify Yin. *Angellicae Gigantis Radix* and *Cnidii Rhizoma* play a role in harmonizing the blood. *Paeoniae Radix* and *Glyzzirizae Radix* harmonize nutrients, and *Pinelliae Tuber* warms the middle part of the body to harmonize the stomach. *Moutan Cortex Radicis* cools the blood, dissipates stasis, and unblocks the meridian. *Zingiberis Rhizoma* and *Evodiae Fructus* warm and tonify the life gate [14]. In particular, *Ginseng Radix Alba* is known for its remarkable adaptogenic quality of helping the body to adapt to cold, fatigue, and stress. Previous trials demonstrated that *Ginseng Radix Alba* considerably enhances the body’s capacity to cope with extreme temperatures, emotional and mental stress, and hunger. *Angellicae Gigantis Radix* is a warming herb that improves blood circulation to the hands, feet, and abdomen [15]. Through the combined effects of these herbs, OKT warms and nourishes the meridian to dissipate cold and is widely used clinically in the treatment of gynecological diseases and coldness in the body. OKT has been reported to have clinical efficacy in the treatment of ovulation disorders, vaginal bleeding, and infertility [16–18]. *Jin kui yao lue*, a textbook of Chinese herbal medicine, states that OKT improves coldness in the lower body of women as well as infertility and ovulation disorders, as mentioned above. One study using a laser Doppler fluxmeter demonstrated that OKT treatment significantly increased peripheral blood flow in the lower extremities of patients with coldness [19]. The OKT granules used in this clinical trial are also recommended for relieving coldness of the lower extremities. According to a Delphi study for the treatment standardization of coldness in the hands and feet, OKT is a commonly used medicine for CHHF in practice in both decoctions and extracts (granules) [6]. Thus, we decided to prescribe OKT to study CHF.

This study has some strengths and limitations. This will be the first RCT to evaluate the efficacy and safety of OKT in treating CHF symptoms. The PubMed database revealed four studies when searched with “*wen jing tang*”. A total of 688 papers were searched on the Chinese database China National Knowledge Infrastructure CNKI using the search word “*温经汤* (*wen jing tang*)” and 127 papers were searched using the search word “*温经汤* (*wen jing tang*) *and 寒* (*coldness*)”. Searching the Korean database OASIS revealed 10 articles which showed the results of domestic research on OKT. There were many studies on the effects of OKT on gynecological diseases and coldness, but there were no RCTs to examine the safety and efficacy of OKT for CHF. This study has another strength: specifically, the team members engaged in this clinical trial are familiar with the SOPs of this study, as they were part of a previous pilot study that investigated the effects of *Danggui-SayukGa-Osuyu-Saenggang-tang* [20], *Ojeok-san* [21], or *Sipjeondaebo-tang* [22] on CHHF.

However, this study has a few limitations. First, a few participants in this trial are likely to be able to distinguish subtle differences between the placebo and test drugs, because they may have been a participant in similar clinical trials at the recruiting hospital. Second, because CHHF is a disease that is easily affected by cold weather, it is possible that changes in weather may have an influence on the outcomes. Therefore, although the BT measurement and the CST are performed after a relaxation period of 20 min at 24 ± 1 °C in the examination room, warmer weather is likely to produce better results, and colder weather may negatively affect the results. Furthermore, we could not fully exclude other underlying diseases that cause CHHF, because laboratory tests such as the antinuclear antibody (ANA) test could not be performed due to the small size of our research grant. Additionally, because the pathogenesis of CHHF is not clear, all of the CHHF-related diseases are not fully known. Finally, this study sets the criteria for dropout, which may affect the results of this study. For example, those who have used medications or treatments that may have effects on the symptoms of CHF, such as anticoagulants and psychotropic drugs, will be withdrawn. Nevertheless, those who take other medications may have taken them because OKT had not improved their symptoms. As such, when we drop out the subjects with low therapeutic effect, it may influence the results of the study. Nevertheless, efficacy data will be analyzed based on the ITT analysis; therefore, dropout is very unlikely to translate into better results, and in general, the more dropouts, the lower the cure rate. Among the forbidden drugs, anticoagulants were contraindicated, because the body temperature can be increased due to the blood flow-improving effects of anticoagulants. Drugs for hyperthyroidism and antipyretic analgesics were prohibited because they can affect the body’s heat metabolism. Psychotropic drugs were prohibited because they may cause problems related to research ethics. In addition, those whose compliance is less than 70% will be withdrawn. During the clinical trial period, compliance should be monitored to guarantee the power of the study. Compliance data are helpful for the interpretation of trial results to avoid erroneous conclusions and to enrich the value of the results [23]. High levels of non-compliance in a clinical trial may provoke the risk of a type II error (also called beta error or manufacturer’s risk). This is the risk of finding no meaningful difference in drug action between the test drug and control when there is a real difference. Increasing the sample size can reduce this risk; however, it will induce an increase of the expense in trial cost [24]. If the compliance is less than 70%, the effect of the OKT formulation can be lowered, and the result of the study may not be accurate; therefore, those with low compliance will be withdrawn. Poor compliance was also suggested as a dropout criterion in other clinical trials studying PURIAM110 (a dietary supplement) [25], Korean red ginseng [26], and Taeumjowi-tang (a traditional Korean medicinal extract) [27], among others. Other requirements are also proposed as dropout criteria because they may affect ethical issues or research accuracy. Although these criteria may have some impact on the outcome of the study, they were presented to comply with research ethics and to verify the effect of the research medication itself.

In spite of these limitations, the current study should add valuable information to the medical literature, as it is the first clinical trial to assess the efficacy and safety of OKT for the treatment of CHF. We anticipate that this study will provide evidence for treating CHF symptoms with the herbal medicine OKT, and that this clinical trial protocol will offer basic data for designing a further large-scale RCT.

### Trial status

The recruitment of participants has been ongoing since 28 May 2018 and will be completed in May 2020. The currently active protocol version is 2.1 (21 May 2018).

## Supplementary information

**Additional file 1.** SPIRIT 2013 checklist.

## Data Availability

The de-identified individual patient data acquired from this study will be available from (handr88@korea.com) on reasonable request in December 2022.

## Abbreviations

AE: Adverse event
ALT: Alanine aminotransferase
ANA: Antinuclear antibody
ANOVA: Analysis of variance
AST: Aspartate aminotransferase
BT: Body skin temperature
BMI: Body mass index
BUN: Blood urea nitrogen
CHF: Cold hypersensitivity in the feet
CHH: Cold hypersensitivity in the hands
CHHF: Cold hypersensitivity in the hands and feet
Cr: Creatinine
CRA: Clinical research associate
CRC: Clinical research coordinator
CRF: Case report form
CRO: Contract research organization
CST: Cold stress test
DBP: Diastolic blood pressure
DMC: Data monitoring committee
r-GTP: Gamma-glutamyl transpeptidase
Hb: Hemoglobin
hCG: Human chorionic gonadotropin
IDMC: Independent Data Monitoring Committee
IP: Investigational product
IRB: Institutional Review Board
ITT: Intention-to-treat
KFDA: Korea Food and Drug Administration
KM: Korean medicine
LOCF: Last-observation-carried-forward
OKT: *Onkyeong-tang*
PI: Principal investigator
PP: Per-protocol
RBC: Red blood cell
RCT: Randomized clinical trial
RP: Raynaud’s phenomenon
RR: Recovery rate
SAE: Serious adverse event
SBP: Systolic blood pressure
SOP: Standard operating procedure
TSH: Thyroid-stimulating hormone
VAS: Visual Analog Scale
WBC: White blood cell
WHOQOL-BREF: World Health Organization Quality of Life Scale-abbreviated version
